# Peruvian validation and standardization of the TabCAT-brain health assessment

**DOI:** 10.3389/fpubh.2025.1600131

**Published:** 2025-08-29

**Authors:** Andrés Muñoz-Najar, Manuel Montemurro, María del Carmen Tejada, Claudia Rivera-Fernández, Miguel Sánchez-Fernández, Nilton Custodio, Katherine L. Possin, Elena Tsoy, Serggio Lanata, Marcio Soto-Añari

**Affiliations:** ^1^Laboratorio de Neurociencia Cognitiva, Universidad Católica San Pablo, Arequipa, Peru; ^2^Escuela de Psicología, Universidad del Alba, Santiago, Chile; ^3^Instituto de Bienestar Socioemocional, Facultad de Psicología, Universidad del Desarrollo, Santiago, Chile; ^4^Center for Social and Cognitive Neuroscience (CSCN), School of Psychology, Universidad Adolfo Ibáñez, Santiago, Chile; ^5^Universidad Tecnológica del Perú, Arequipa, Peru; ^6^Clinica del Sur, Arequipa, Peru; ^7^Instituto Peruano de Neurociencia, Lima, Peru; ^8^Memory and Aging Center, Department of Neurology, Weill Institute for Neurosciences, University of California San Francisco, San Francisco, CA, United States; ^9^Global Brain Health Institute (GBHI), University of California, San Francisco, San Francisco, CA, United States

**Keywords:** Alzheimer’s disease, mild cognitive impairment, cognitive assessment, brief cognitive tests, regression-based norming, digital cognitive tools, diagnostic accuracy, low educational attainment

## Abstract

**Introduction:**

Detecting cognitive impairment in low-educated and marginalized populations may result in under- or over-estimation of diagnoses due to reliance on non-validated approaches and normative data. This study validates and standardizes TabCAT-BHA for older adults living in the Andean region of Peru using regression-based normalization.

**Methods:**

Two hundred fifty-eight participants were assessed with the MMSE, RUDAS, and TabCAT-BHA. Classified as either cognitively healthy or impaired based on Clinical Dementia Rating criteria.

**Results:**

By incorporating sex, place of residence, age, and years of education as covariates, the TabCAT-BHA demonstrated greater accuracy in detecting cognitive impairment (AUC = 75.3%) compared to the MMSE (AUC = 66.4%) and RUDAS (AUC = 71.4%). After incorporating only significant sociodemographic predictors, TabCAT-BHA obtained better AUC (77.4%) compared to MMSE (66.6%) and RUDAS (71.9%).

**Discussion:**

The TabCAT-BHA proves to be a valid tool for detecting cognitive impairment, and incorporating sociodemographic factors improves its accuracy in marginalized settings of Peru.

## 1 Introduction

As the global population ages at an unprecedented rate, the prevalence of age-related degenerative brain diseases, such as Alzheimer’s disease (AD), is rising worldwide. These diseases are characterized by a gradual decline in cognitive function. Affected individuals first experience mild cognitive impairment (MCI), a precursor to the more severe stage of illness known as dementia, which involves significant cognitive decline and loss of functional abilities. In AD, MCI often precedes the onset of dementia by several years.

The global prevalence of dementia of all causes has grown significantly, from 20.3 million cases in 1990 to 43.8 million in 2016, with projections reaching 152 million by 2050 (1). This increase represents a substantial burden not only for those directly affected but also for caregivers, families, and society at large (2). Therefore, it is important to detect degenerative brain diseases early, before the dementia stages of illness, while affected individuals are still in MCI stages. Early detection and diagnosis promote improved quality of life through timely prognostic and supportive interventions, pharmacologic and non-pharmacologic treatment optimizations, and other approaches to personalize the longitudinal care of these chronic diseases (3).

Diagnosing AD in MCI stages of illness is more important than ever, as rapidly emerging disease-modifying therapies for AD are effective only during MCI to early dementia stages, before cognitive decline is severe, and functional abilities are decisively compromised (4). It is, therefore, imperative that primary care providers become comfortable diagnosing AD in MCI stages, in preparation for increasing widespread access to these emerging therapies globally.

The accurate detection of MCI is challenging, however, partly owing to a lack of highly sensitive and specific brief cognitive tests (BCTs) that can be applied in diverse sociodemographic settings (5). One of the most used BCTs is the Mini-Mental State Examination (MMSE), but this BCT is limited by its inadequate longitudinal reliability and its limited sensitivity in detecting mild cognitive changes (6). Additionally, research suggests that the performance of the MMSE may be influenced by sociodemographic factors such as age, cultural background, and educational level, which restricts its applicability in certain population groups (7).

Other BCTs have been developed seeking to overcome these limitations. One such BCT is the Rowland Universal Dementia Assessment Scale (RUDAS), which has proven to be a useful test in low-resource settings, as it exhibits limited bias in individuals with little or informal education and requires minimal cultural or linguistic adaptations (8). In Peru, however, it has been observed that illiterate individuals living in rural areas score significantly lower on this test compared to their urban peers (9), suggesting that contextual factors such as access to education and the sociocultural environment significantly influence its applicability.

In primary care settings, the ideal BCT should efficiently detect early signs of cognitive decline and provide accurate assessments of key cognitive domains and functional abilities (to distinguish MCI from dementia) while adapting to contextual factors (10). Therefore, it is necessary to explore new BCTs that allow for a more precise identification of common cognitive syndromes in diverse contexts.

Digitally based BCTs, such as the Tablet-Based Cognitive Assessment Tool Brain Health Assessment (TabCAT-BHA), have been shown to be more accurate and efficient than traditional paper-based assessments (11). This BCT represents a significant advance in primary care, as the TabCAT-BHA assesses different cognitive domains and incorporates stimuli and response formats adapted to diverse cultures and educational levels, making it accessible and effective in various contexts (12). In the primary care setting, this BCT not only reduces the time and costs associated with cognitive testing but also optimizes diagnostic accuracy through automated scoring systems, producing immediate evidence-based assessments. As an automated tool, it has been observed to significantly reduce the time needed for administration, scoring, and interpretation, which implies cost reduction and optimal use of available time and human resources. Additionally, it helps reduce disparities in access to care, as it is available in multiple languages (11).

The TabCAT-BHA has shown higher sensitivity for detecting early symptoms of neurocognitive disorders, and better long-term stability compared to traditional paper-based measures (10, 13, 14). Similarly, its effectiveness has been confirmed in the Latin American population for the early detection of MCI in low- and middle-income countries (15), benefiting healthcare providers by optimizing diagnostic accuracy and simplifying the care process (16).

Moreover, the TabCAT-BHA is amenable to regression-based norming that can adjust for demographic and/or social factors, which can improve accuracy of detection over traditional binning procedures or using the same cut-point for all individuals (17–19). Regression-based norming allows the creation of norms better suited and adapted to individual variations in demographic and social characteristics (i.e., sex, age, place of residence, years of education, etc.). With this approach, researchers and clinicians are able to draw more accurate and context-sensitive trajectories for participants and patients.

This study sought to validate the TabCAT-BHA in a sample of Peruvian adults with low educational levels from rural and urban areas. We included healthy individuals and participants with MCI. This validation, using a regression-based approach, represents a key opportunity to advance early detection and management of cognitive decline in contexts with high levels of educational and sociocultural diversity.

## 2 Materials and methods

### 2.1 Participants

The study sample, acquired through non-probabilistic convenience sampling (Table 1), consisted of 258 older adults between the ages of 54 and 91 years from the Andean city of Arequipa, located in the southern region of Peru. The sample included adults under 60 years old, assuming that normal cognitive decline can start as early as age 50 or the chance of dementia young-onset (20, 21). Participants came from the rural district of Pampacolca, located at an altitude of 2,916 meters above sea level, and from the urban district of Arequipa, located 2,328 meters above sea level. Most of them (69.38%) were bilingual (Quechua-Spanish). All participants were assigned a clinical diagnosis [cognitively healthy (Control) or mild cognitive impairment (Case)] based on a gold-standard clinical diagnostic protocol independent of the results of the BCT instruments included in the analyses. Due to the low number of participants in the case group, no distinction was made between MCI subtypes. Exclusion criteria included severe psychiatric disorders, other non-neurodegenerative neurological disorders, substance use disorders, and significant vision or hearing limitations.

**Table 1:** Participant characteristics.

	n/M	%/SD
Sex		
Male	91	35.27
Female	167	64.73
Residency		
Rural	172	66.67
Urban	86	33.33
CDR category		
Case (CDR = 0.5 and CDR = 1)	72	27.91
Control (CDR = 0)	186	72.09
Age	69.50	8.79
Formal education (years)	3.44	2.29

*n*, frequency; M, mean; SD, standard deviation; CDR, clinical dementia rating.

### 2.2 Instruments

The TabCAT-BHA (10) was developed at the Memory and Aging Center of the University of California San Francisco (UCSF). The TabCAT-BHA assesses different cognitive domains through four subtests (for an example see Supplementary Figure S1): The Favorites subtest (associative memory domain), Match subtest (processing speed and executive functions domain), Line Orientation subtest (visuospatial skills domain), and Animal Fluency subtest (language domain). Testing time is approximately 12 min. The tasks’ descriptions, Spanish language adaptation, and other psychometric properties of the TabCAT-BHA are available on their website.^1^

The Peruvian version of the MMSE (22) and RUDAS tests (9) also assess different cognitive domains. The MMSE assesses five cognitive domains: orientation, registration, attention and calculation, recall, and language. The RUDAS assesses six cognitive domains: immediate memory, visuospatial orientation, motor praxis, visuospatial construction, judgment, recent episodic memory, and language. Both BCTs have been studied and widely used in diverse Peruvian settings.

### 2.3 Procedure

The study received approval from the Ethics Committee of the Directorate of Research (CEDI) at Universidad Católica San Pablo (Act 002. CEDI. UCSP.2020 from July 2, 2020). All participants provided written informed consent. The study protocol ensured data privacy and confidentiality while minimizing risks to participants. Participants were informed of their right to withdraw from the study at any time.

All participants were assessed with a previously described gold-standard clinical assessment protocol, including a complete neuropsychological assessment and a clinical interview (23) [for more detail, see Rivera-Fernández et al. (23)]. Participants were categorized according to the CDR scoring system (see Table 1). The CDR assessment was administered by trained research assistants and one of the authors (CR-F), a neuropsychologist formally trained and certified in the application of the CDR scoring system. Additionally, the CDR scores were reviewed by a panel of clinicians which include a neuropsychologist (MS-A) and a neurologist (NC). For the TabCAT-BHA administration, all participants used a 9.7-inch iPad, in a horizontal position. For the other measurements, the application followed the standardized protocol for each one.

### 2.4 Data analysis

We analyzed data obtained from the CDR, demographic variables, MMSE, RUDAS, and TabCAT-BHA scores. The statistical analyses were conducted using the R programming language with RStudio as the Integrated Development Environment (IDE). The specific packages used are listed in Supplementary material. Following previously described methodology (14), we applied a regression-based norming approach to the raw scores of the TabCAT-BHA, MMSE, and RUDAS. This approach adjusts each score for demographic and other variables (e.g., age, sex, education, and residence), enabling their interpretation relative to a normative population. Age and education were modeled as continuous variables, while sex (0 = male, 1 = female) and residence (0 = rural, 1 = urban) were modeled as binary. For the TabCAT-BHA, a composite score (BHA-CS) was calculated by summing the weighted demographically adjusted subtests based on the logistic regression analyses as previously described (14). For MMSE and RUDAS, the adjusted scores were derived by adjusting the total raw score on each test for demographic variables. Predictor variables were selected based on both theoretical considerations (variables expected to be related based on literature but not necessarily showing strong statistical significance) and empirical evidence, we have provided Supplementary Tables S1–S3 detailing these analyses (variables demonstrating significant correlations, as shown in Supplementary Table S1). Two analyses were performed, one using all predictors, and the other one using only statistically significant predictors. For the logistic regression models, we set the significance threshold at *p* < 0.10 to reduce the risk of Type II errors (false negatives), given that the aim instance is detecting true effects.

### 2.5 Norming procedure

Following the guidelines in Tsoy et al. (14), we based our analysis on the results of the Animal Fluency (language), Favorites (associative memory), and Match (executive functioning and processing speed) subtests. The Line Orientation (visuospatial skills) was not included in the analysis due to prior evidence of its non-significant contributions to discriminating against Controls and Cases (14). The first step involved calculating the regression coefficients for the demographic variables using data only from the cognitively healthy participants (Supplementary Tables S2, S3). We performed multiple linear regression analyses with each subtest score as the outcome and each demographic variable as a predictor. We found that the residuals of regression models for the Animal Fluency and Match subsets had a non-normal distribution, and the residuals of regression model for the Match test had heteroskedasticity; a bootstrap correction and HC3 heteroskedasticity correction were used, respectively, yet after these corrections the regression coefficients remained unchanged.

After the weighted *Z*-scores were calculated, we performed two logistic regression models to calculate the TabCAT-BHA composite score (BHA-CS). The first model (Table 2) used the weighted *Z*-scores for all predictors (Supplementary Table S2). The second model (Table 3) used the weighted *Z*-scores for only significant predictors (Supplementary Table S3). In both models, the dependent variable was the diagnosis (0 = Control, 1 = Case).

**Table 2:** Logistic regression model to determine weightings for BHA-CS calculation based on weighted *Z*-scores of all predictors.

	*B*	SE	*Z*-value	*p*	OR
Intercept	−1.364	0.18	7.53	< 0.001	
Animal fluency *Z*	−0.436	0.16	2.73	0.006	0.647
Favorites *Z*	−0.578	0.15	3.78	< 0.001	0.561
Match *Z*	−0.358	0.17	2.06	0.040	0.699

*B*, unstandardized coefficient; SE, standard error; OR, odds ratio.

**Table 3:** Logistic regression model to determine weightings for BHA-CS calculation based on weighted *Z*-scores of significant predictors.

	*B*	SE	*Z*-value	*p*	OR
Intercept	−1.542	0.20	7.65	< 0.001	
Animal fluency *Z*	−0.457	0.16	2.83	0.005	0.633
Favorites *Z*	−0.673	0.16	4.20	< 0.001	0.510
Match *Z*	−0.335	0.17	1.92	0.055	0.715

*B*, unstandardized coefficient; SE, standard error; OR, odds ratio.

Two receivers operating characteristic (ROC) analyses were performed, the first ROC curve used the coefficients from Table 2, including significant and non-significant sociodemographic predictors. The second ROC curve used coefficients from Table 3, including only significant predictors.

## 3 Results

Once the regression coefficients were calculated for the cognitively healthy group, a weighted *Z*-score for each subtest was calculated for all participants (cognitively healthy and cases), including significant and non-significant predictors, using the following Equation 1 (for details see Supplementary Tables S4, S5):


(1) $$\begin{array}{l} \mathrm{Zw}=\\ \left(\frac{\mathrm{Raw}\;\text{Score}-(B_{0}+B_{1}*\mathrm{Age}+B_{2}*\mathrm{Sex}+B_{3}*\text{Education}+B_{4}*\text{Residency})}{\text{Residual Standard Error}}\right) \end{array}$$

Then, the weighted *Z*-scores of each subtest were introduced into a logistic regression model, where the outcomes were the cognitively healthy or case (MCI) assignments. To calculate the BHA-CS, first, we obtained unstandardized raw BHA-CS scores, as seen in Equation 2, with *B*’s absolute values from the logistic regression results:


(2) $$\begin{array}{l} \mathrm{Raw}\;\mathrm{BHA}-\mathrm{CS}=B_{0}+B_{1}*\text{Zanimal}+B_{2}*\text{Zfavorites}\\ +B_{3}*\text{Zmatch} \end{array}$$

Then, we calculated the mean and standard deviation scores of the raw BHA-CS in cognitively normal subjects only and derived the final standardized BHA-CS as follows Equation 3 (for the final calculation see Supplementary Table S6):


(3) $$\mathrm{BHA}-\mathrm{CS}=(\frac{\mathrm{Raw}\;\mathrm{BHA}-\mathrm{CS}-{\bar{X}}_{\text{RawBHA}-\text{CSCN}}}{SD_{\text{RawBHA}-\text{CSCN}}})$$

For the MMSE and RUDAS total raw scores, we calculated the regression coefficients for sex, age, education, and residency, computed a weighted *Z*-score, and calculated the final *Z*-score. The second analysis included multiple regression models only including predictors that were individually significantly correlated with raw scores.

The same approach was used for calculating the new weighted *Z*-scores. In the final weighted *Z*-scores, the Animal Fluency subtest was weighted by age, sex, and years of education; the Favorites subtest was weighted by residency; and the Match subset was weighted by age, years of education, and residency. For the MMSE, the predictors used were age and years of education, and for the RUDAS, we used age and years of education. We repeated the logistic regression analyses to derive the BHA-CS with updated weights from individual TabCAT-BHA subtests adjusted only for significant predictors.

### 3.1 ROC curves

**Figure 1:**
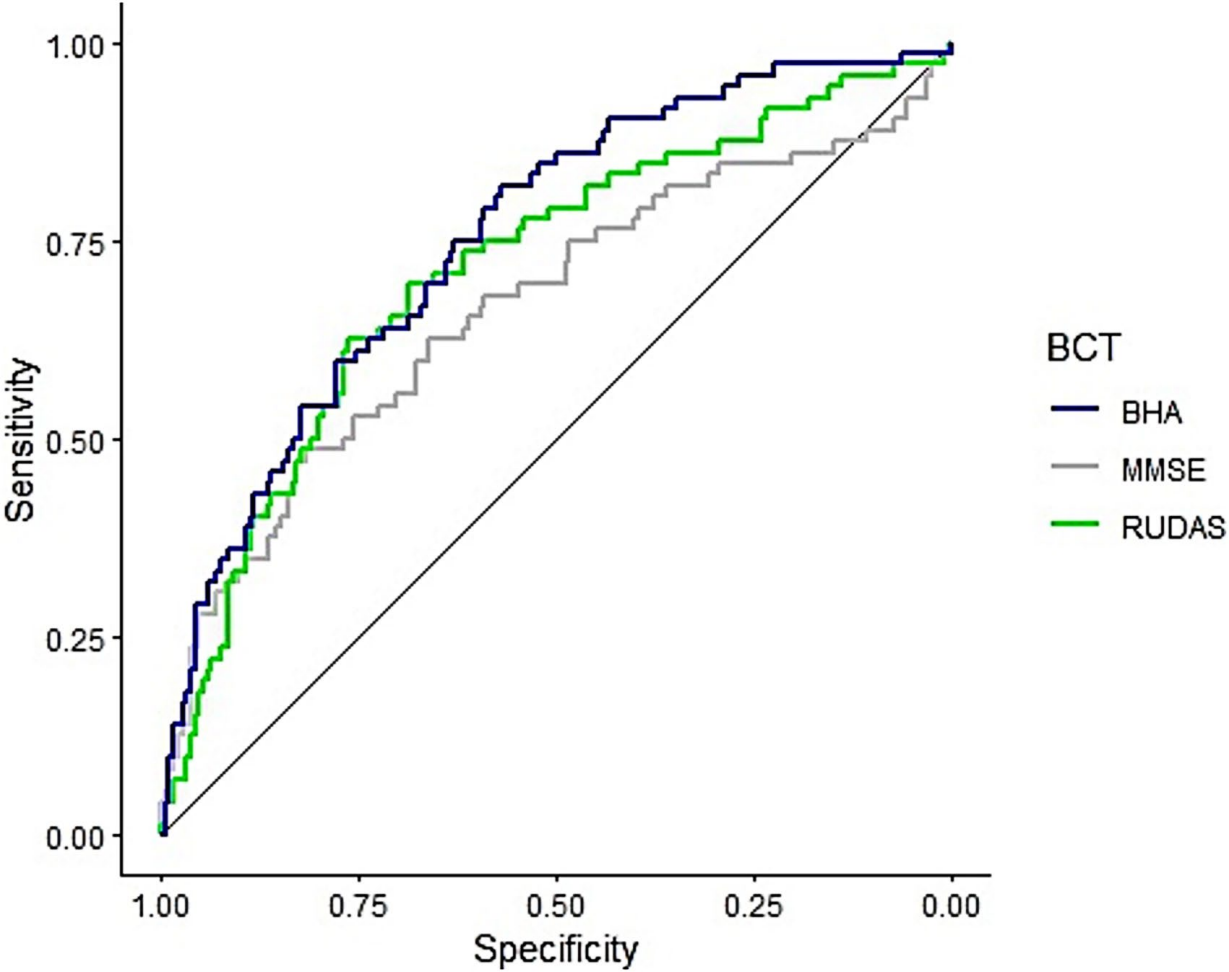
ROC curves for the TabCAT-BHA, MMSE, and RUDAS adjusted for both significant and non-significant sociodemographic predictors. BHA, Brain Health Assessment; MMSE, Mini-Mental State Examination; RUDAS, Rowland Universal Dementia Assessment Scale.

Two ROC analyses were performed, comparing the BHA-CS with MMSE and RUDAS weighted scores. For the first ROC curve we used the weighted scores of both significant and non-significant sociodemographic predictors, and for the second one we used only significant sociodemographic predictors. The results (Figure 1) indicate BHA-CS’s (Specificity = 57.0%, Sensitivity = 81.9%, Accuracy = 63.95%) AUC captures 75.3% of positive cases, against the MMSE’s 66.4% (Specificity = 81.7%, Sensitivity = 48.6%, Accuracy = 72.5%) and RUDAS’s 71.4% (Specificity = 76.3%, Sensitivity = 62.5%, Accuracy = 72.5%).

**Figure 2:**
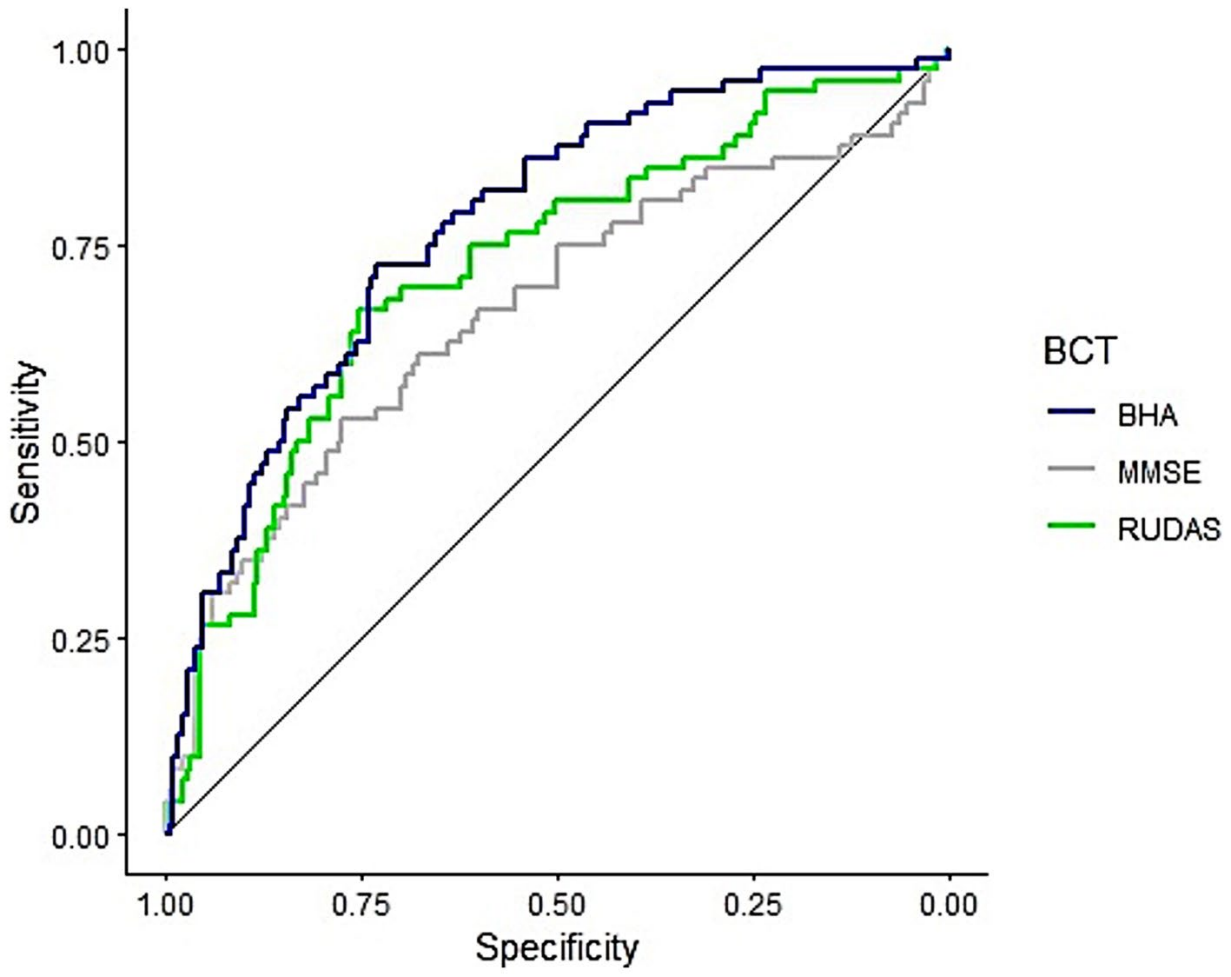
ROC curves for the TabCAT-BHA, MMSE, and RUDAS adjusted only for significant sociodemographic predictors. BHA, Brain Health Assessment; MMSE, Mini-Mental State Examination; RUDAS, Rowland Universal Dementia Assessment Scale.

In the second (Figure 2) ROC curve (Specificity = 73.1%, Sensitivity = 72.2%, Accuracy = 72.9%) the resulting AUC captures 77.4% of the positive cases against the MMSE’s 66.6% (Specificity = 77.4%, Sensitivity = 52.8%, Accuracy = 70.5%) and RUDAS’s 71.9% (Specificity = 75.3%, Sensitivity = 66.7% Accuracy = 72.9%).

In both cases (with and without non-significant predictors), the BHA-CS showed better performance compared to its traditional counterparts. In the first ROC curve, BHA-CS reported significantly better AUC than MMSE (*D* = −1.91, *p* = 0.028), and better but not significantly than RUDAS (*D* = −1.16, *p* = 0.123). In the second ROC curve, BHA-CS reported significantly better AUC than MMSE (*D* = −2.46, *p* = 0.007), and better but not significantly than RUDAS (*D* = −1.25, *p* = 0.105).

Finally, in a secondary analysis, a BHA-CS’s categorization was created and associated with the CDR scores. For the BHA-CS’s categorization, cutoff points were established: scores above −0.99 indicated cognitively healthy participants, scores between −1.99 and −1.00 were participants with MCI, and scores below −2.00 would be participants with possible dementia, considering the traditional ±2 SD cutpoint. A cross-table (Table 4) between BHA-CS’s categorization and CDR showed minor variations in the classification (*χ*^2^ = 32.58, *p* < 0.001). Notably, among the 26 participants whom CDR diagnosed as cognitively healthy, the BHA-CS categorization identified 23 cases as MCI and 3 as Possible Dementia. With the second BHA-CS score (including adjustment for only significant predictors), similar variations in the classification (*χ*^2^ = 35.43, *p* < 0.001) were observed. Consistently, among the same 26 participants whom CDR diagnosed as cognitively healthy, this second BHA-CS score also characterized 23 cases as MCI and 3 as Possible Dementia. These results show how empirical sociodemographic variables can affect the MCI and dementia test scores and diagnosis.

**Table 4:** Cross table BHA-CS’s categorizations and CDR.

	Control	Case	Total	*χ*^2^(2)
All sociodemographic covariables				32.58***
CH (> − 0.99)	160	39	199	
MCI (−1.99; −1.00)	23	24	47	
P-DM (< −2.00)	3	9	12	
Only significant sociodemographic covariables				35.43***
CH (> − 0.99)	160	37	197	
MCI (−1.99; −1.00)	23	28	51	
P-DM (< −2.00)	3	7	10	

CH, cognitive healthy; MCI, mild cognitive impairment; P-DM, possible dementia.

***p < 0.001.

## 4 Discussion

The results of the study indicate that the TabCAT-BHA is a valid tool for assessing cognitive health in low-educated older adults living in rural and urban settings of Arequipa, Peru. Furthermore, it demonstrates higher accuracy in identifying cognitively healthy individuals compared to those with mild cognitive impairment. In addition, the TabCAT-BHA demonstrates superior performance compared to other widely used brief cognitive assessment tools in Peru, such as the MMSE and RUDAS.

Using a regression-based norming approach for the TabCAT-BHA allows clinicians and researchers to estimate patients’ and participants’ performance more accurately. This approach offers a crucial advantage: the ability to automate the incorporation of covariates (e.g., age, sex, education) into the analysis and calculate standardized, weighted scores that account for the effects of these covariates (14, 17, 18).

Unlike traditional norming methods, which rely on predefined rankings and procedures, the regression-based approach could reduce the mean and median differences across score ranges, especially in age-adjacent ranges (19). This enables more nuanced and equitable comparisons of individuals’ performance by adjusting for demographic factors that may influence test outcomes. An additional advantage of regression-based norming approaches is their ability to achieve accurate results with smaller sample sizes (19). Traditional range-based norming methods typically require a large sample size to establish reliable norms, which can be time- and cost-intensive and prohibitive in low-resource settings. In contrast, regression-based norming allows clinicians and researchers to achieve precision with fewer participants, making it a more efficient and cost-effective approach (19).

Reliable normative scores are essential to monitor age-related cognitive changes. Researchers and clinicians must have accurate methods to monitor the progression of participants and patients over time. The use of traditional normative ranges often lacks the sensitivity needed to detect subtle changes over time (19, 24), thus limiting their effectiveness in tracking the trajectory of cognitive decline. In this context, the findings reported in this study, particularly the high sensitivity achieved by scores adjusted for demographic and sociocultural variables, enable the detection of subtle variations in cognitive performance. These variations can help identify early symptoms (i.e., MCI) associated with degenerative brain diseases such as AD. Furthermore, the TabCAT-BHA’s integration of tests that assess multiple cognitive domains—such as memory, speed, and executive functions—enables the detection of early specific deficits. For example, impairments in associative memory or processing speed, captured by the TabCAT-BHA, may represent early indicators of underlying degenerative brain diseases (10, 11, 13, 14).

The TabCAT-BHA is a robust tool, both clinically and psychometrically, for evaluating cognitive impairment. Additionally, it addresses various concerns faced by healthcare professionals, particularly in primary healthcare settings. The validation conducted in this study, employing regression techniques, provides a more effective response to the concerns raised by Sideman et al. (16) regarding the limited experience of primary care healthcare personnel in cognitive evaluations, as well as the challenges in delivering diagnoses influenced by various context-specific variables of the individuals being assessed.

On the other hand, most experienced clinicians and researchers agree that BCTs alone are insufficient when evaluating cognitive impairment, and that an accurate diagnosis requires a more nuanced and comprehensive approach (16, 25). However, having a valid assessment tool is a key component of a diagnostic evaluation, and having an efficient digital tool could help to optimize and economize the limited resources available. This is of utmost importance in limited-resource settings such as those frequently encountered in Latin American countries.

Considering the high rates of illiteracy or low educational attainment in Latin America, this BCT provides an opportunity to achieve more accurate assessments in socioeconomically vulnerable and diverse populations. In this context, our study findings support the TabCAT-BHA as a tool with high sensitivity and specificity indices, outperforming the most commonly used assessments in Peru and Latin America, such as the MMSE and RUDAS. Moreover, it enables clinical professionals to achieve standards comparable to those in other Latin American countries (15) and developed nations (10, 14, 26), using validated and standardized assessments (27).

However, it is important to note that the specificity of the TabCAT-BHA was lower in the initial model (ROC 1), which included both significant and non-significant sociodemographic covariates, compared to the MMSE and RUDAS. This result may be due to the inclusion of theoretical or irrelevant variables that introduced noise into the model, thereby increasing false positives. In contrast, the second model (ROC 2), which retained only significant sociodemographic covariates, showed improved specificity and classification accuracy. This finding highlights the importance of including only meaningful predictors in regression-based standardization, as doing so enhances the precision of cognitive classification and reduces misclassification due to demographic noise.

The findings of the study support the TabCAT-BHA’s utility in Arequipa. While the application of its weighted *Z*-scores to other regions within Peru is a crucial consideration, their broader applicability is likely, given similarities across the country, particularly given the shared challenges in educational quality and access, especially in rural areas like Pampacolca (7, 9). Nevertheless, future standardization studies incorporating samples from diverse Peruvian regions would further enhance the TabCAT-BHA’s discriminability, specificity, sensibility, and overall accuracy.

However, the current norming rules may not be transferred to other countries. This is primarily due to the significant heterogeneity in sociocultural and educational characteristics that distinguish other nations from Peru. However, the use of regression-based norming with weighted *Z*-scores offers a notable advantage: it facilitates the comparison of scores with those from other countries that have also employed regression-based norming using the same covariates. This methodological consistency allows for broader cross-cultural comparisons, even when direct application of specific norms is not feasible.

The results obtained in our sample reveal a notable discrepancy between the diagnostic classification provided by the Clinical Dementia Rating (CDR) and that derived from the TabCAT-BHA, contributing to the broader discussion regarding the limitations of the CDR as a primary diagnostic tool. In our case, the CDR classified 186 participants as cognitively healthy (CDR = 0) and 72 as having some degree of cognitive impairment (CDR = 0.5 and 1). However, when applying the TabCAT-BHA algorithm, the distribution shifted, identifying 199 participants as cognitively healthy and only 59 as impaired. This divergence may reflect previously noted methodological limitations, noting that the Clinical Dementia Rating (CDR) was originally designed as a staging instrument rather than a diagnostic tool, and that its reliability depends heavily on clinical judgment and the subjective integration of functional and cognitive information (28). Moreover, the global scoring method based on a “dominance rule” may obscure domain-specific discrepancies and does not necessarily reflect the patient’s true cognitive profile (29).

In this context, the TabCAT-BHA offers substantial advantages by incorporating demographically corrected, automated neuropsychological tasks that assess memory, language, executive functioning, emotional status, and daily functioning. The use of regression-based adjustments for age, sex, education, and residency allows for more accurate and equitable classification, particularly in diverse populations. In contrast to the CDR, which applies a dominance-based scoring rule that may inadequately capture symptom heterogeneity, the TabCAT-BHA provides a quantitative and reproducible approach to detecting cognitive impairment, thereby reducing both false positives and subjective clinical bias (16). In this regard, our findings support the use of the TabCAT-BHA as a more sensitive, specific, and operationally feasible alternative for early cognitive impairment detection in primary care settings, while the CDR may remain valuable for longitudinal staging in contexts with greater access to expert clinical interpretation.

A significant limitation of this study is the cross-sectional nature of its design, which does not allow for the evaluation of the TabCAT-BHA’s performance in tracking cognitive decline over time. This limitation highlights the need for future research that incorporates longitudinal follow-up. A longitudinal study of the TabCAT-BHA would enable the examination of its sensitivity to assess change across different stages of cognitive decline, providing a more accurate measure of disease progression in specific patients. Another limitation is that the diagnoses were based on clinical and neuropsychological data, but did not include laboratory, neuroimaging, genetic or pathological data, as well as biomarker testing. Another limitation was that MCI patients were grouped together as a single category, without distinguishing between amnestic and non-amnestic subtypes. Future standardization studies should account for this heterogeneity to ensure more accurate and representative normative data. With respect to sample heterogeneity, bilingualism is a common characteristic in Andean populations. In this study, it was measured dichotomously and not included as a covariate due to the language-independent nature of the TabCAT-BHA. Nonetheless, future research should examine bilingualism as a potential predictor—considering both its presence and degree of use as a continuous variable—given its known impact on cognitive functioning.

Finally, this study introduces a valuable tool for the Peruvian healthcare system. Specifically, it offers evidence of an automated tool that facilitates the evaluation process in non-specialty settings. By reducing the time required for administration, scoring, and interpretation, it contributes to cost reduction and optimization of the limited temporal and human resources available, particularly in primary healthcare settings (11).

As an automated BCT, the TabCAT-BHA is also uniquely positioned to support the development of a database for iteratively tracking research participants’ and patients’ cognitive health. Such a database could help identify early symptoms of cognitive decline, guide and monitor complex cognitive health evaluations, and inform interventions aimed at preventing or slowing cognitive decline. Moreover, this database would provide healthcare systems with more precise epidemiological data, thereby enhancing public policy decision-making regarding cognitive health and treatment strategies.

In conclusion, this study aimed to clinically validate and standardize the brief digital TabCAT-BHA in Peruvian older adults living in urban and rural settings. By applying a novel approach to standardize psychological and neuropsychological tests, the TabCAT-BHA represents an innovative BCT that enables clinicians and researchers to detect cognitive decline in early stages. The composite score, adjusted for demographic and sociocultural covariates, strengthens the precision and reliability of cognitive health assessments. Finally, the TabCAT-BHA has the potential to transform the processes of cognitive health assessment, treatment, and guidance.

## Data Availability

The raw data supporting the conclusions of this article will be made available by the authors, without undue reservation, to any qualified researcher upon request.

## Footnotes

^1^ tabcathealth.com
