# Supplementary material for: Peruvian validation and standardization of the TabCAT-brain health assessment
Source: Front Public Health. 2025 Aug 29;13:1600131. doi: 10.3389/fpubh.2025.1600131 (PMC12425762; doi:10.3389/fpubh.2025.1600131)
Supplement: Supplementary file 1 [file Data_Sheet_1.docx]

Supplementary Material

# Supplementary Figures and Tables

## Supplementary Figures

## Figure S1

## *TabCAT-BHA’s Subtests Examples for the Peruvian Standardization*

##
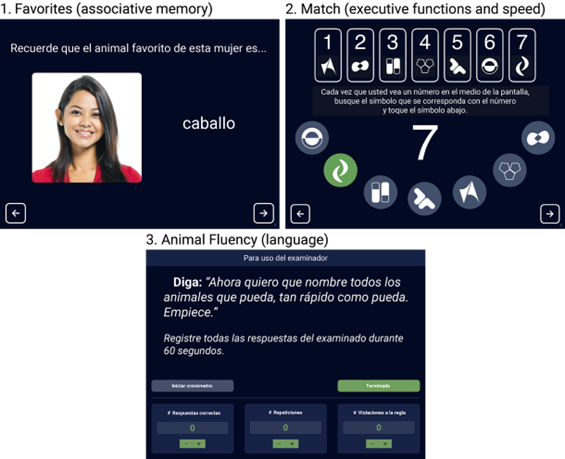


## *Note*. 1: Favorites subtest; 2: Match subtest; 3: Animal Fluency subtest

## Figure S1 shows one example of each TabCAT-BHA’s subtests, used in Peruvian’s standardization. Favorites assess associative memory, where the participant must remember the picture’s favorite food or animal. Scores range from 0 to 24, where greater values indicates better performance. Match is a subtest designed to assess executive function and processing speed. It involves participants identifying pairs of stimuli as quickly as possible. The score on this subtest is based on the number of correct answers and the response time. Animal Fluency is a subtest designed to assess semantic verbal fluency, an essential component of language, by asking the participant to name as many animals as possible in a limited period of time, usually 60 seconds.

## Supplementary Tables

**Table S1**

*Correlations among cognitive assessments and sociodemographic variables*

|  | Sex^1^ | Residency^2^ | Age | Years of education |
| --- | --- | --- | --- | --- |
| MMSE | -.22** | .08 | -.45*** | .79*** |
| RUDAS | -.14 | -.09 | -.37*** | .54*** |
| Animal Fluency | -.25*** | .00 | -.29*** | .42*** |
| Favorites | -.16* | -.39*** | -.09 | .09 |
| Match | .00 | .45*** | -.26*** | .28*** |

*Note*. * *p* < .05, ** *p* < .01, *** *p* < .001. ^1^Reference group = Male. ^2^Reference group = Rural

Pearson correlations were calculated among MMSE, RUDAS, TabCAT-BHA’s subtests and sociodemographic variables (i.e. sex, residency, age, and years of education). Sex (male = 0, female = 1) and residency (rural = 0, urban = 1) were modelled as binary. Age and years of formal education were modelled as continuous variables.

Sex shows significant negative correlations with MMSE, Animal Fluency, and Favorites, that means males performed worse than females in those measurements. Residency shows negative significant correlation with Favorites, but positive with Match, that means people who live in urban places perform better in Match, but worse in Favorites. Age and years of formal education correlate with MMSE, RUDAS, Animal Fluency, and Match. However, age has negative correlations and years of formal education have positive ones.

**Table S2**

*Regression models with all predictors in cognitively healthy participants*

|  | *B* | *SE* | *t value* | *p* |
| --- | --- | --- | --- | --- |
| *Animal Fluency* |  |  |  |  |
| Intercept | 20.281 | 2.27 | 8.926 | < .001 |
| Age | -0.081 | 0.028 | -2.867 | .005 |
| Sex^1^ | -1.288 | 0.487 | -2.643 | .009 |
| Education (years) | 0.45 | 0.11 | 4.083 | < .001 |
| Residency^2^ | -0.194 | 0.48 | -0.405 | .686 |
| *Favorites* |  |  |  |  |
| Intercept | 15.838 | 2.44 | 6.492 | < .001 |
| Age | -0.053 | 0.03 | -1.742 | .083 |
| Sex^1^ | -0.769 | 0.523 | -1.469 | .144 |
| Education (years) | 0.082 | 0.118 | 0.695 | .488 |
| Residency^2^ | -2.96 | 0.515 | -5.748 | < .001 |
| *Match* |  |  |  |  |
| Intercept | 29.968 | 7.05 | 4.254 | < .001 |
| Age | -0.185 | 0.088 | -2.106 | .037 |
| Sex^1^ | -0.411 | 1.511 | -0.272 | .786 |
| Education (years) | 0.871 | 0.342 | 2.549 | .012 |
| Residency^2^ | 9.629 | 1.487 | 6.474 | < .001 |
| *MMSE* |  |  |  |  |
| Intercept | 28.184 | 1.05 | 26.92 | < .001 |
| Age | -0.057 | 0.013 | -4.396 | < .001 |
| Sex^1^ | -0.225 | 0.225 | -1.002 | .317 |
| Education (years) | 0.727 | 0.051 | 14.323 | < .001 |
| Residency^2^ | -0.024 | 0.221 | -0.107 | .915 |
| *RUDAS* |  |  |  |  |
| Intercept | 29.365 | 1.02 | 28.678 | < .001 |
| Age | -0.044 | 0.013 | -3.416 | < .001 |
| Sex^1^ | -0.061 | 0.22 | -0.277 | .782 |
| Education (years) | 0.346 | 0.05 | 6.967 | < .001 |
| Residency^2^ | -0.537 | 0.216 | -2.486 | .014 |

*Note*. ^1^Reference group = Male. ^2^Reference group = Rural

Table S2 shows regression coefficients for each TabCAT-BHA’s subtests, MMSE, and RUDAS. All predictors, including non-significant ones, were entered into the models. The decision was taken for theoretical reasons. All models were corrected with 2000 bootstrap samples, because some models violated the normality or homoscedastic assumption. The Animal Fluency test shows a decline in performance with age and poorer performance in men, while education is associated with significant improvement. However, residence has no significant effect on this test. In the Favorites test, neither age, sex nor education have a significant influence, but the residential environment does show an impact, with poorer performance in participants from rural areas. The Match test is also negatively affected by age, but improves with more years of education and with residence in urban environments. The MMSE reveals that age reduces performance while education improves it, but neither sex nor residence has a significant impact. In the RUDAS test, age decreases performance and education improves it, although a slight effect of residence is observed, with lower scores in people from rural areas.

**Table S3**

*Regression models with significant predictors in cognitively healthy participants*

|  | *B* | *SE* | *t value* | *p* |
| --- | --- | --- | --- | --- |
| *Animal Fluency* |  |  |  |  |
| Intercept | 20.209 | 2.26 | 8.942 | < .001 |
| Age | -0.080 | 0.028 | -2.854 | .005 |
| Sex^1^ | -1.316 | 0.481 | -2.734 | .007 |
| Education (years) | 0.446 | 0.109 | 4.072 | < .001 |
| *Favorites* |  |  |  |  |
| Intercept | 12.466 | 0.409 | 30.479 | < .001 |
| Sex^1^ | -0.788 | 0.492 | -1.601 | .111 |
| Residency^2^ | -2.806 | 0.514 | -5.456 | < .001 |
| *Match* |  |  |  |  |
| Intercept | 29.227 | 6.481 | 4.51 | < .001 |
| Age | -0.179 | 0.085 | -2.104 | .037 |
| Education (years) | 0.903 | 0.319 | 2.829 | .005 |
| Residency^2^ | 9.572 | 1.468 | 6.518 | < .001 |
| *MMSE* |  |  |  |  |
| Intercept | 28.176 | 1.04 | 27.071 | < .001 |
| Age | -0.057 | 0.013 | -4.41 | < .001 |
| Sex^1^ | -0.229 | 0.222 | -1.031 | .304 |
| Education (years) | 0.726 | 0.05 | 14.416 | < .001 |
| *RUDAS* |  |  |  |  |
| Intercept | 28.904 | 0.946 | 30.557 | < .001 |
| Age | -0.040 | 0.013 | -3.17 | .002 |
| Education (years) | 0.345 | 0.047 | 7.333 | < .001 |

*Note*. ^1^Reference group = Male. ^2^Reference group = Rural

Table S3 The cognitive tests evaluated in the TabCaT-BHA show differences in their sensitivity to different key factors. The Animal Fluency test is negatively affected by age and sex, with poorer performance in men, while education acts as a protective factor. The Favorites test does not show a significant impact of age, but does show a difference according to residence, with poorer performance in rural areas. Regarding the Match test, its performance decreases with age but improves with more years of education, in addition to showing an advantage for those living in urban environments. The global evaluation using the MMSE and the RUDAS reveals a decrease in performance with age, but both tests show a positive effect of education, suggesting that the level of schooling can partially compensate for the cognitive decline associated with aging. Taken together, these results highlight the influence of age and education on cognitive performance, as well as the impact of the environment on certain specific skills.

**Table S4**

*Weighted Z scores with all predictors*

| **Test** | ***z*-score** |
| --- | --- |
| Match | (Raw score - (29.968 - 0.185*Age - 0.411*Male + 0.871*Education + 9.629*Rural))/9.085 |
| Favorites | (Raw Score - (15.838 - 0.053*Age - 0.769*Male + 0.082*Education - 2.960*Rural))/3.146 |
| Animal Fluency | (Raw score - (20.281 - 0.081*Age - 1.288*Male + 0.450*Education - 0.194*Rural))/2.930 |

*Note*. Age = Age in years, Male = Male sex (0), Education = Formal education in years, Rural = Rural residency (0)

Table S4 shows weighted Z scores with regression coefficients applying the formula 1 in the main paper.

**Table S5**

*Weighted Z scores with significant predictors*

| **Test** | ***z*-score** |
| --- | --- |
| Match | (Raw score - (29.227 - 0.179*Age + 0.903*Education + 9.572*Urban))/9.062 |
| Favorites | (Raw score - (12.466 - 2.806*Rural))/3.174 |
| Animal Fluency | (Raw score - (20.209 - 0.080*Age - 1.316*Male + 0.446*Education))/2.923 |

*Note*. Age = Age in years, Male = Male sex (0), Education = Formal education in years, Rural = Rural residency (0)

Table S5 shows weighted Z scores with regression coefficients applying formula 1 in the main paper. However, this formula only uses significant predictors from regression models.

**Table S6**

*Algorithm for standardized TabCAT-BHA-CS*

| **Version** | **Formula** |
| --- | --- |
| All predictors | ((1.364 + 0.436 * *Z*_Animal_ + 0.578 * *Z*_Favorites_ + 0.358 * *Z*_Match_) - 1.36)/0.92 |
| Significant predictors | ((1.542 + 0.457 * *Z*_Animal_ + 0.673 * *Z*_Favorites_ + 0.335 * *Z*_Match_) - 1.43)/1.00 |

Table S6 shows both algorithms for TabCAT-BHA composite scores. First, using all theoretical predictors, and second using only significant predictors, from the regression models.
